# Identification and Linkage to Care of HCV-Infected Persons in Five Health Centers — Philadelphia, Pennsylvania, 2012–2014

**Published:** 2015-05-08

**Authors:** Catelyn Coyle, Kendra Viner, Elizabeth Hughes, Helena Kwakwa, Jon E. Zibbell, Claudia Vellozzi, Deborah Holtzman

**Affiliations:** 1 National Nursing Centers Consortium; 2 Philadelphia Department of Public Health; 3 Division of Viral Hepatitis, CDC

Approximately three million persons in the United States are infected with hepatitis C virus (HCV), a blood-borne pathogen that is an increasing cause of liver disease and mortality in the United States (1,2). Treatments for HCV are curative, of short duration, and have few associated side effects (3), increasing the importance of identifying HCV-infected persons. Many persons with HCV infection were infected decades ago, before implementation of prevention measures and most are unaware of their infection, regardless of when it occurred (4). Most newly diagnosed cases are associated with injection drug use (5). Persons born during 1945–1965 have a fivefold higher risk of HCV infection than other adults and the highest risk for HCV-related morbidity and mortality (6). CDC recommends testing for this group, for persons who inject drugs, and others at risk for HCV infection (6,7). From October 2012 through July 2014, the National Nursing Centers Consortium (NNCC) carried out a project to integrate routine HCV testing and linkage-to-care in five federally qualified health centers in Philadelphia, PA, that primarily serve homeless persons and public housing residents. During the project period, 4,514 patients across the five centers were tested for HCV. Of these, 595 (13.2%) were HCV-antibody positive and 550 (92.4%) had a confirmatory HCV-RNA test performed. Of those who had a confirmatory HCV-RNA test performed, 390 (70.9%) were identified as having current (i.e., chronic) HCV infection (overall prevalence = 8.6%). Of those currently infected with HCV, 90% were informed of their status, 78% were referred to an HCV care specialist, and 62% went to the referred specialist for care. Replicable system modifications that improved HCV testing and care included enhancements to electronic medical records (EMRs), simplification of HCV testing protocols, and addition of a linkage-to-care coordinator. Findings from this project highlight the need for innovative strategies for HCV testing, care, and treatment, as well as the important role of community health centers in expanding access for patient populations disproportionately affected by HCV infection (1).

In 2012, the NNCC, a national membership organization functioning to advance nurse-led care, partnered with its parent company, Public Health Management Corporation (PHMC)*, to implement routine HCV testing and referral to care in PHMC’s five federally qualified health centers (FQHCs) in Philadelphia, PA: 1) Mary Howard Health Center (exclusively serving homeless patients); 2) Rising Sun Health Center and 3) PHMC Health Connection (both family medicine clinics serving public housing residents); 4) Congreso Health Center (serving primarily Hispanic patients); and 5) PHMC Care Clinic (offering primary care and specialized health services to patients with unmet medical and social needs, including treatment for patients with human immunodeficiency virus (HIV) and HCV infection). All of these health centers integrated routine HCV testing through a medical assistant-initiated, opt-out, laboratory-based model with EMR modifications to prompt, track, report, and facilitate reimbursement for HCV tests.

Before testing began, the NNCC project manager and a local hepatitis C expert trained clinic personnel on HCV disease etiology, effects, and testing goals. The project manager assisted clinic staff with integrating testing into the existing clinic infrastructure to minimize disruption in routine services. Informational posters were placed in each health center to educate patients on prevalence, risk factors for HCV infection and recommendations for who should be tested. Patients eligible for testing included those born during 1945–1965 (i.e., “Baby Boomers”), those with other risks for HCV infection (e.g., injection drug use) (6,7), and those who were homeless. An automatic electronic reminder, (the first of four EMR enhancements), identified patients eligible for testing based on birth year. Mary Howard Health Center and PHMC Care Clinic tested all patients, because most of those seen at these two clinics were assumed to be at increased risk for HCV infection. At the other three sites, medical assistants interviewed patients and tested those with at least one identified risk factor for HCV infection.

Medical assistants notified patients that they would be tested for HCV unless they opted out. For patients who verbally agreed to be tested, a standing order was in place to initiate the requisition for HCV-antibody with reflex to an HCV-RNA test to detect current HCV infection. With reflex testing, the laboratory uses the same specimen to perform an HCV-RNA test on any positive HCV-antibody test specimen, thus eliminating the need for a second blood specimen to be collected from the patient. NNCC negotiated competitive pricing with commercial laboratories to perform HCV tests on uninsured patients and an account was created and added to the EMR (second EMR enhancement). Selecting this account generated a separate invoice specifically for HCV tests performed on uninsured patients.

Weekly reports showing the number of patients, by health center, who were tested and the names of those whose results were HCV-antibody-positive and HCV-RNA-positive were generated by the EMR (third EMR enhancement). These reports were sent to the project manager who provided the information to the clinic directors, medical assistants, and linkage-to-care coordinator. The latter assisted all patients who were HCV-antibody-positive through the care process, which included providing all current HCV-infected patients with their test results, offering onsite posttest counseling, and referring patients to HCV-care specialists (primary-care providers trained to care for patients infected with HCV, as well as hepatologists or gastroenterologists from one of the local academic medical centers) for medical evaluation. An automatic reminder was generated by the EMR (final EMR enhancement) alerting health-care providers that an HCV-infected patient was eligible for linkage-to-care services, such as an escort to follow-up medical appointments, transportation reimbursements, reminder phone calls, and appointment scheduling. This report reflects data extracted from the EMR that was shared across all five health centers.

From October 1, 2012 through July 31, 2014, a total of 4,514 patients were tested for HCV across the five FQHCs; 595 (13.2%) had a positive HCV-antibody test result (Table). Among the HCV-antibody positive-patients, 550 (92.4%) had an HCV-RNA test performed and of these, 390 (70.9%) were identified with current HCV infection, for an overall prevalence of 8.6%. Most HCV-infected patients were male, non-Hispanic black, and had public insurance compared with others in these demographic subgroups. Although non-Hispanic blacks accounted for a greater proportion of persons with current infection than non-Hispanic whites (53.6% versus 29.5%), non-Hispanic whites had a greater overall prevalence of HCV infection compared with non-Hispanic blacks (21.1% versus 7.3%). Baby Boomers accounted for 62.6% of patients with current HCV infection. Among 352 patients reporting drug use, 205 (58.2%) reported having injected drugs in their lifetime. The two clinics conducting HCV testing for all patients, without ascertainment of risk (the PHMC Care Clinic and the Mary Howard Health Center), performed a greater number of tests compared with the other sites and had the highest proportion of patients who were HCV-antibody-positive and currently infected. Of the 390 persons with current HCV infection, 348 (89.2%) received their HCV-RNA positive results, 304 (78.0%) were referred to an HCV-care specialist for medical evaluation, and 240 (61.5%) were seen by the specialist (Figure). Of the 390 patients with current HCV infection, 25 (6.4%) began antiviral treatment during the data collection period of this project. The PHMC Care Clinic identified 247 HCV-infected patients and successfully linked 167 (67.6%) to medical care. Among the five FQHCs, the PHMC Care Clinic had the highest rates of linkage to care.

During the course of the project, HCV testing and care for patients at the FQHCs improved, as the result of a change in HCV-RNA test availability and additional system modifications. In March 2013, all commercial laboratories began conducting reflex testing. Before this, testing for HCV-RNA was more cumbersome, requiring a second blood specimen be obtained at the same or at a subsequent visit. In the 5 months before reflex testing was routinely available, only 83.6% of the clinics’ patients with positive HCV antibody tests received confirmatory testing. From March through July 2013, early in the transition to reflex testing, this percentage increased to 84.8%. However, by July 2014, as use of the reflex test became more routinely used, the percentage of HCV-antibody-positive patients who received confirmatory testing increased to 96.3%.

Another system modification initiated during the project was a change from provider-initiated testing to medical assistant-initiated testing at the PHMC Care Clinic. At the beginning of the project, a nurse identified and reminded providers (with a chart note) of patients who were eligible for testing and the need to order an HCV test. An EMR prompt to identify Baby Boomers also was in place at this time. However, the process proved time intensive and inefficient: in clinics serving large patient populations with complex needs, chart notes were often overlooked and nurses had many other responsibilities. Medical assistant-initiated HCV testing resulted in a 6.3% increase in testing from an observation period of 11 months before compared with 11 months after implementing this procedural modification.

Beginning in September 2013, through funding from a public-private partnership, annual HIV testing for patients aged ≥13 years was implemented in the health centers, at which time the HCV testing protocol was modified to include HIV testing. Dual testing substantially increased the total number of HCV tests performed. A total of 1,786 HCV tests were performed during the 11-month project period leading up to this modification, whereas from September 2013 through July 2014, a total of 2,728 HCV tests were performed, representing a 52.7% increase.

A final modification that led to improved patient care was the addition of a linkage-to-care coordinator position. This coordinator was responsible for providing intensive services, including contacting patients who did not keep their appointments and addressing any barriers to care (e.g., affordable transportation). Between the 11 months before the addition of the position and 11 months afterwards, these services increased the number of HCV-infected patients who received their positive HCV-RNA test results by 67.7% (from 130 to 218 patients); patient referrals by 49.2% (from 122 to 182); and the number of patients seen by an HCV care specialist by 28.6% (from 105 to 135).

## Discussion

This project demonstrated that routine HCV testing can be successfully integrated into ambulatory care settings providing services for persons disproportionately affected by HCV infection. The project introduced six practices that could be replicated in other clinical settings. First, it tasked medical assistants with guiding patients through the HCV testing process, relieving the burden on clinicians and other health center staff. Second, reflex HCV testing technology was used to ensure that HCV-antibody positive patients received HCV-RNA testing necessary to detect current HCV infection. Such testing also allowed patients to receive test results and care referrals in one visit. Third, test costs associated with patients without insurance were eliminated as a barrier to testing. Fourth, modifying the HCV testing protocol to include HIV testing led to substantial increases in the number of HCV tests performed and currently infected patients identified (82.6% increase). Because more patients were eligible for HIV testing, the HCV test was easily added to the laboratory requisition.

Fifth, EMR modifications also improved patient care. The EMR prompted testing and the need for linkage-to-care services, all of which were monitored by the project manager and the linkage-to-care coordinator. Weekly reports tracked testing and patient progression through the HCV care continuum. EMR modifications also simplified the payment process for HCV tests performed on uninsured patients. Finally, intensive services carried out by the linkage-to-care coordinator increased the number of currently infected patients who received their results and were referred and seen by a specialist. This effort ensured that more patients received appropriate posttest counseling and medical evaluation for liver health and HCV treatment initiation.

The project successfully targeted patients at high risk for HCV infection; overall 8.6% of patients were infected with HCV, a higher proportion than previous estimates. Only 1% (0.8%–1.2%) of the general U.S. population is estimated to be infected with HCV (1). A geographically targeted community-based testing program also carried out in Philadelphia found that 2.8% of persons were estimated to be living with current HCV infection (8). The percentage of patients receiving an HCV-RNA test confirming their current infection status likewise was higher (92% versus 47%) than that reported by the Philadelphia Department of Public Health from routine surveillance of viral hepatitis (9). The higher percentage in this project is largely attributable to the use of reflex testing (10), which ensures that a greater number of persons are tested for current infection and can learn their infection status without returning to provide a second blood specimen.

A relatively small proportion of HCV-antibody-positive patients (7.6%) did not receive a confirmatory test during the project period. There are several possible reasons for this. Some patients submitted specimens early in the project, before the implementation of reflex testing, and might not have returned for confirmatory HCV-RNA test. Specimens submitted for confirmatory testing might not have met laboratory testing requirements because of insufficient quantity or improper handling. Providers might have inadvertently ordered the hepatitis panel that currently only includes an HCV-antibody test.

Linkage-to-care rates also were higher than those observed in Philadelphia surveillance data (9), an outcome likely attributed to creation of the linkage-to-care coordinator position. Initially, linkage services included reminder phone calls, public transportation tokens for patients to attend appointments, or patient escorts. However, some patients were found to require additional services. The linkage-to-care coordinator provided intensive support services: following up with patients who did not keep appointments; conducting off-site (e.g., home, shelters, and halfway houses) visits as necessary; acting as an intermediary point of contact between the patient and the FQHCs; and helping identify and resolve any barriers patients experienced in attending appointments. These services fostered trusting relationships with patients. Additionally, the linkage-to-care coordinator remained a point of contact for HCV-infected patients that had fallen out of care because of addiction, unstable housing, or distrust of health care systems. The linkage-to-care coordinator worked with these patients until they were ready to reengage in care, linked them to social and addiction support programs, and provided specialized care plans to ensure they attended their HCV medical appointments. Similar intensive linkage services in community-based HCV testing and linkage-to-care programs in Philadelphia also have proven successful in navigating HCV-infected patients into care (8).

The most successful linkage-to-care rates were seen at the PHMC Care Clinic, where HCV testing, care, and treatment are provided in the same setting. This test-and-treat model eliminates the need to refer patients to an outside care provider, except in extenuating circumstances (e.g., a patient with advanced liver disease or cirrhosis). Because of the high number of HCV-infected patients seen there, the Mary Howard Health Center plans to expand its services to include on-site HCV treatment. Linkage-to-care rates were lowest at health centers serving patients at low risk of HCV infection. Because they served fewer HCV-infected patients, providers at these sites might not be as aware of the HCV linkage-to-care protocol. To increase rates in these settings, HCV protocols will be updated and included in an automated centralized forum accessible by staff for training.

What is already known about this topic?

Hepatitis C virus (HCV) infection is a major cause of chronic liver disease and hepatocellular carcinoma and the leading indication for liver transplantation in the United States. Approximately three million persons in the United States are infected with HCV, and many are unaware of their status and are diagnosed late. As persons disproportionately affected by HCV (e.g., poor, homeless, born during 1945–1965, injection drug users) age, HCV-related morbidity, mortality, and spending are expected to increase.

What is added by this report?

Routine HCV testing and linkage-to-care for persons with current infection were successfully integrated into five federally qualified health centers in Philadelphia, PA. Across the centers, 595 (13.2%) of 4,514 patients tested were HCV-antibody positive and 550 (92.4%) received HCV-RNA testing. Of the 390 (70.9%) with current (chronic) HCV infection, 348 (89.2%) received their results, 304 (78.0%) were referred to and 240 (61.5%) were seen by a provider familiar with HCV care and treatment. This project demonstrated the feasibility of identifying low-income persons living with HCV infection and linking them to care.

What are the implications for public health practice?

In collaboration with public health agencies and other service providers, community health centers are optimally positioned to play a pivotal role in expanding access to recommended HCV testing, care, and treatment for populations disproportionately affected by hepatitis C. Delivering HCV-related care via trusted healthcare professionals, with the addition of embedded and intensive support services, at primary care settings can increase successful outcomes at every stage of the care continuum.

There were two main limitations of this project. The first was its relatively small size. HCV testing and linkage to care were integrated into a small network of health centers and therefore the practices and lessons learned from the project may not be applicable to larger settings, such as a large hospital system, or in other geographic areas. To address this, NNCC is exploring ways to replicate this model in larger health center networks in other cities. Second, the duration of the project did not follow patients through HCV treatment to cure. NNCC is working with offices of local HCV providers to collect data on treatment history and clinical outcomes.

The high rate of HCV infection among persons in disproportionately affected populations, like those seen at the five FQHCs, indicates a need for innovative models that can identify persons with HCV infection and ensure they receive appropriate care. Delivering care via trusted health care professionals at primary care settings can improve outcomes at every stage of the continuum of care, from reflex testing to providing timely test results and linking patients to HCV-focused care. In collaboration with public health agencies and other service providers, community health centers are optimally positioned to play an important role in expanding access to recommended HCV testing, care, and treatment for populations disproportionately affected by hepatitis C.

## Figures and Tables

**FIGURE:**
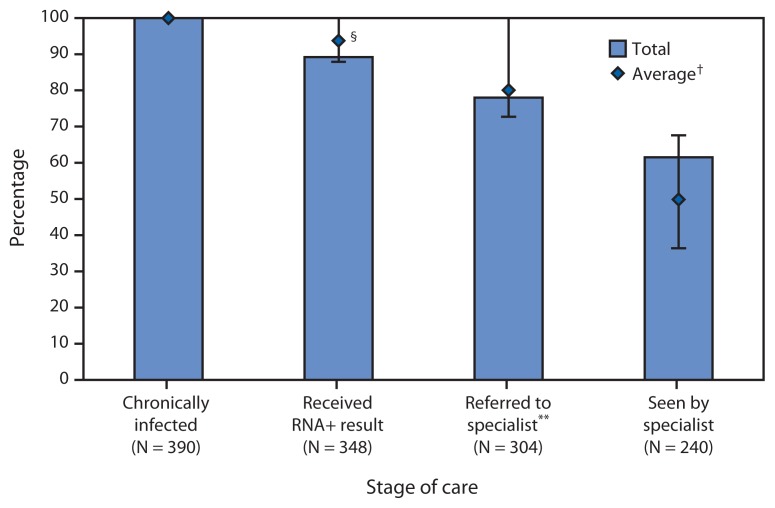
Contiuum of care process for patients with chronic hepatitis C (HCV) infection^*^ treated at five federally qualified health centers (FQHCs)^†^ — Philadelphia, PA, October 2012–July 2014 **Abbreviation:** RNA+ = Patients whose specimens tested positive for HCV. ^*^ Patients with chronic HCV infection are defined as those who are currently infected with HCV based upon a positive result to HCV-RNA test. ^†^ All five FQHCs are owned and managed by Philadelphia-based Public Health Management Corporation. ^§^ Error bars are the range of percentages for each stage of care across all five FQHCs. ^¶^ Average = average of values at all five FQHCs. ^**^ Specialists include primary care providers who were trained to care for patients infected with HCV, as well as hepatologists or gastroenterologists from one of the local academic medical centers.

**TABLE:** Number, percentage, and prevalence of patients tested for HCV, and identified as HCV-antibody positive and currently infected*, by demographic characteristics and health centers† — Philadelphia PA, October 2012–July 2014

Characteristic	HCV-Antibody Tested		HCV-Antibody Positive			Currently Infected§		
		
	No.	(%)	No.	(%)¶	Prevalence** (%)	No.	(%)††	Prevalence§§ (%)
**Sex**								
Male	2,522	(55.9)	421	(70.8)	(16.7)	297	(76.2)	(11.8)
Female	1,992	(44.1)	174	(29.2)	(8.7)	93	(23.8)	(4.7)
**Race/Ethnicity**								
Non-Hispanic Black	2,862	(63.4)	309	(51.9)	(10.8)	209	(53.6)	(7.3)
Non-Hispanic White	545	(12.1)	173	(29.1)	(31.7)	115	(29.5)	(21.1)
Hispanic	724	(16.0)	81	(13.6)	(11.2)	48	(12.3)	(6.6)
Asian	136	(3.0)	5	(0.8)	(3.7)	5	(1.3)	(3.7)
Other	77	(1.7)	2	(0.3)	(2.6)	1	(0.3)	(1.3)
Missing	170	(3.8)	25	(4.2)	(14.7)	12	(3.1)	(7.1)
**Birth Year Cohort**								
<1945	53	(1.2)	2	(0.3)	(3.8)	2	(0.5)	(3.8)
1945–1965	1,890	(41.9)	366	(61.5)	(19.4)	244	(62.6)	(12.9)
>1965	2,571	(57.0)	227	(38.2)	(8.8)	144	(36.9)	(5.6)
**Health Insurance Type**								
Uninsured	1,495	(33.1)	126	(21.2)	(8.4)	77	(19.7)	(5.2)
Public Insurance	2,704	(59.9)	433	(72.8)	(16.0)	290	(74.4)	(10.7)
Private Insurance	315	(7.0)	36	(6.1)	(11.4)	23	(5.9)	(7.3)
**Health Center**								
Care Clinic	1,518	(33.6)	358	(60.2)	(23.6)	247	(63.3)	(16.3)
Mary Howard	1,079	(23.9)	159	(26.7)	(14.7)	108	(27.7)	(10.0)
PHMC Health	837	(18.5)	31	(5.2)	(3.7)	11	(2.8)	(1.3)
Connection								
Rising Sun	808	(17.9)	24	(4.0)	(3.0)	12	(3.1)	(1.5)
Congreso	272	(6.0)	23	(3.9)	(8.5)	12	(3.1)	(4.4)
**Total**	**4,514**	**(100)**	**595**	**(100)**	**(13.2)**	**390**	**(100)**	**(8.6)**

**Abbreviation:** HCV = hepatitis C virus

* Currently infected indicates a diagnosis of chronic infection with HCV based on positive results of HCV-RNA testing.

† Health centers are five federally qualified health centers owned and managed by the Public Health Management Corporation, Philadelphia, PA.

§ 550 (92.4%) of 595 persons with HCV-antibody positive tests received HCV-RNA testing.

¶ Percent positive among those tested.

** Percent positive among those tested in each demographic subgroup and health center.

†† Percent currently infected among those tested.

§§ Percent currently infected among those tested in each demographic subgroup.

## Footnotes

* The Public Health Management Corporation (PHMC) is a nonprofit public health institute that builds healthier communities through partnerships with government, foundations, businesses and community-based organizations (additional information available at http://www.phmc.org/site/index.php).
